# Best Practice Principles to Work With Consumer Representatives on Patient Safety Investigation Teams

**DOI:** 10.1111/hex.70543

**Published:** 2026-01-23

**Authors:** Yinghua Yu, Charlotte J. Molloy, Lorelle Bowditch, Mia Bierbaum, Liat Watson, Jennifer Morris, Zoe Fernance, Jenny Berrill, Duncan Brown, Matthew Ames, Peter D. Hibbert

**Affiliations:** ^1^ Australian Institute of Health Innovation, Faculty of Medicine, Health and Human Sciences Macquarie University Macquarie Park New South Wales Australia; ^2^ Independent Consumer Advisor; ^3^ IIMPACT in Health, Allied Health and Human Performance University of South Australia Adelaide South Australia Australia

**Keywords:** adverse event, adverse events reviews, best practice principles, consumer representative, patient safety, patient safety investigation

## Abstract

**Introduction:**

Consumer representatives (CRs), individuals with lived experience and other consumer advisers and volunteers, play an increasing role in advancing consumer‐oriented healthcare. Some health services have begun integrating CRs into patient safety investigation teams with the aim of ensuring patient and family perspectives are incorporated into the process. Recent empirical research has begun to shed light on the perceptions and experiences of CRs in these roles. Built on these insights, this paper proposes a set of best practice principles for the effective engagement of CRs in patient safety investigations.

**Methods:**

This study adopted a qualitative approach, drawing on data collected through interviews with 11 CRs and 10 focus groups with health service staff from Victoria, Australia. A co‐design workshop was conducted with an advisory committee of six consumers from four Australian jurisdictions. Thematic analysis was employed to analyse the data and identify key themes.

**Results:**

Four high‐level themes emerged: (1) formalise the CR role, including the implementation of a standardised recruitment process with defined professional and personal selection criteria to ensure an appropriate fit for the role, as well as appropriate remuneration and considerations for inclusion and equity; (2) investigation team‐level support, providing structured training for CRs, chairs and other team members to consider the team dynamics and interactions as a whole. Form buddy/mentorship programmes to ensure consistent engagement and support processes; (3) organisational integration, embedding the CR role within the organisational structure, including dedicated escalation pathways, ongoing evaluation and feedback mechanisms for continuous improvement of the role; (4) system‐wide cultural shift, promoting the recognition and valuing CRs through committed leadership, legislative support and awareness building at all levels of the organisational hierarchy, and developing a shared pool of trained CRs.

**Conclusion:**

The paper proposes best practice principles to optimise the engagement of CRs on patient safety investigation teams.

**Patient or Public Contribution:**

Eleven individuals with experience as CRs on patient safety investigation teams were interviewed. Additionally, six consumers from four Australian jurisdictions—members of a research grant consumer advisory committee and co‐authors of this paper—collaborated on the study design, contributing to data interpretation and writing, and worked with the research team to develop the proposed best practice principles.

## 1 Introduction

### 1.1 Background

In recent years, there has been increasing recognition that healthcare systems can cause serious harm to patients through adverse events [1, 2]. Globally, adverse events are a major concern for patient safety and the quality of care [3, 4], with approximately 1 in 10 hospital admissions linked to such events [5]. Examples of these events are medication errors (such as incorrect medications or dosages) [6], healthcare‐associated infections (e.g., post‐surgical infections), and delays in identifying and responding to a patient's physical or mental deterioration [7]. In some cases, adverse events can result in long‐term physical disability or psychological trauma, and unexpected death [8, 9, 10].

When an adverse event resulting in serious harm to a patient occurs, health services generally establish an investigation team (also called review teams) to examine what happened, identify contributing factors and recommend changes to prevent similar adverse events in the future [11, 12, 13]. These teams typically consist of clinical experts, safety and quality personnel, and, in some cases, human factors specialists [8, 14]. Recently, there has been increasing interest in the potential benefits of including consumer representatives (CRs) on these teams to foster a more balanced understanding of events [15] and ensure that investigation outcomes better reflect the needs of patients and families [16]. Unlike other consumer engagement in advisory groups, which often involves long‐term engagement, patient safety investigation teams operate in a time‐limited (usually 60–90 days), high‐intensity context to review a single adverse event and then dissolve once the investigation is completed [17].

The public health system in the Australian state of Victoria is among the first to formally introduce an expectation that a CR will be on patient safety investigation teams [8, 16]. This development builds on a long history of consumer advocacy in Australia, with co‐design approaches more formally recognised over the past decade in healthcare services [18, 19]. From a national policy perspective, consumer partnership is written into the national standard for ensuring the quality of health services and the safety of patients in hospitals [20]. The role of CRs on investigation teams includes advocating for a patient‐centred approach, challenging assumptions, identifying overlooked issues and contributing to public trust through greater accountability and transparency, whilst remaining independent from the event [16]. Our previous research has shown that CRs significantly enhance the focus on patients and their families during investigations [15]. This study of CRs on patient safety investigation teams suggests that CRs' engagement is heavily influenced by the chair/facilitator of the team, and the CR's contribution to the outcomes of reviews is generally positive, a view also supported by health service staff [15].

### 1.2 Aims

In research that we have previously published, we undertook interviews and focus groups with CRs and health service staff that explored the perceptions and experiences of CRs involved in patient safety investigations [15]. This current paper aims to present a set of best practice principles for effectively engaging CRs on investigation teams based on the consultations with CRs and staff, with an additional co‐design process with a consumer advisory committee.

## 2 Methods

### 2.1 Research Design

This paper employs a qualitative research approach, utilising data gathered from focus groups with health service staff and semi‐structured interviews with CRs, and then a co‐design workshop with a consumer advisory committee. The study was approved by the Northern Sydney Local Health District (NSLHD) Human Research Ethics Committee (approval number: 2023/ETH02431). This research is part of a wider programme of research for a National Health and Medical Research Council Partnership Grant that aims to improve the healthcare system's response when patients experience harm [21]. This paper is guided by the Consolidated Criteria for Reporting Qualitative (COREQ) checklist [22] (see Appendix 1).

### 2.2 Setting

Public health services in the state of Victoria, Australia, participated in this study. Victoria accounts for roughly 39% (6.5 million) of the Australian population [23], with its health services including public hospitals, regional health services and community care. Within this system, investigation teams are usually convened by local health networks or hospital services according to state‐level clinical governance policies and guidance from Safer Care Victoria [14].

### 2.3 Participants

This study involved CRs and multidisciplinary health service staff who have worked alongside CRs in these teams. The co‐design workshop involved an advisory committee of six consumers.

### 2.4 Recruitment

A purposive sampling approach was adopted to recruit participants with experiences in patient safety investigations across Victorian public health services. Study participants were invited to participate by Health Service Directors of Clinical Governance/Safety and Quality or Safe Care Victoria (SCV) via a formal email invitation with a link to a RedCap participant information and consent form. RedCap is a secure web platform for building and managing research online databases and surveys (https://redcap.mq.edu.au/). Concurrently, a survey (see Appendix 2) was conducted to collect demographic information, including participants' role (e.g., CR, clinicians, governance and so forth), gender, jurisdiction and experience with patient safety investigation teams. Following completion of the consent and demographic information, the research team contacted the participant to organise a time for the interview and/or focus group.

### 2.5 Data Collection

The research team conducted one‐to‐one interviews with CRs, to allow the sharing of in‐depth personal experiences [24], and focus groups with health services staff to encourage peer discussion and collective reflections about their roles and experiences with investigation teams [25]. Interviews and focus groups were conducted by the lead researcher P.H. and Y.Y. via Microsoft Teams between April and July 2024. The primary data source was interviews with CRs, conducted using semi‐structured schedules (Appendix 3) developed from the patient safety literature [10], with a focus on CRs' perceptions and experiences of their roles, including benefits, challenges, risks and the training and support required. Focus groups with health services staff were primarily centred on their experiences within investigations more broadly [17], with supplementary questions also asked around their experiences working with CRs on investigation teams; these were collected to provide a complementary perspective to the CRs. (focus group guide, Appendix 4).

### 2.6 Consumer Advisory Committee

Members of the consumer advisory committee were recruited purposively through professional and consumer networks associated with the project partner Investigators. They were recruited from four Australian jurisdictions: Victoria, New South Wales, Queensland and the Australian Capital Territory. All members have at least 5 years of experience in consumer roles within health care, with some having over a decade of involvement. Their role in the broader project is consultative, providing feedback across the project to reflect the rigour and relevance for consumers, and includes meeting at least quarterly with the research team for the life of the project. For this research, they participated in a co‐design workshop to assist with the interpretation of results and provide feedback on the best practice principles. The workshop was conducted via Teams in August 2024 (co‐design workshop guide, Appendix 5).

### 2.7 Data Analysis and Interpretation

All interviews and focus groups lasted between 45 min and 1 h. Audio recordings were transcribed using Microsoft Teams' automated transcription feature and subsequently verified for accuracy by Y.Y. (research fellow, PhD, female). The verified transcripts were then uploaded to NVivo 14 for thematic analysis [26] using an inductive reasoning approach.

**Figure 1:**
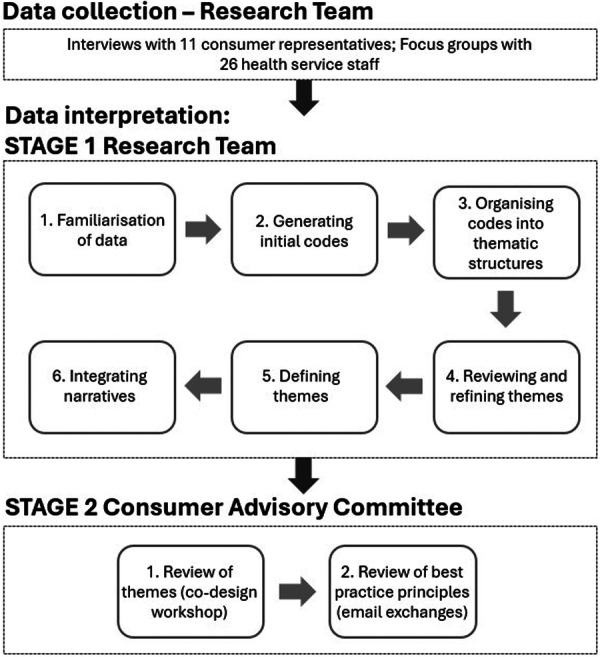
Data collection and analysis.

Data interpretation was conducted across two stages by the research team and the consumer advisory committee (see Figure 1). In the first stage, the research team engaged in detailed discussions to identify and refine emerging themes from the interviews and focus groups. Initially, the research team familiarised themselves with the data through repeated reading of the transcripts and note‐taking. Next, the initial codes (e.g., CR needs, benefits/risks and support) were generated inductively to capture CRs' involvement in investigation teams. Codes were then organised in thematic structures (e.g., major themes and minor themes) and discussed with the team for consensus. These themes were reviewed and refined for clarity and relevance. Themes were then defined and supported with quotes. Finally, narratives were integrated with the interpretation of existing literature and policy frameworks. Analysis was primarily led by Y.Y., who has a sociological background and experience in qualitative studies, with weekly discussions with the lead researcher P.H. and team, who are experts in patient safety and human factors, to ensure reflexivity and relevance.

At the second stage, the research team presented the findings to the consumer advisory committee and invited feedback. In total, two rounds of feedback were sought. In the first round, feedback was shared during the workshop, and some committee members' reflexive insights in working on investigation teams were also incorporated into the findings. This collaborative discussion was thematically analysed and shaped into four overarching categories, which formed the foundation for developing best practice principles for engaging CRs in patient safety investigations. In the second round, the research team drafted a set of best practice principles based on these themes and emailed them to the committee for feedback. These principles were refined further in response to their comments to ensure clarity, relevance and practical applicability. This co‐design methodological approach has been effective for consumer engagement in the existing literature [27, 28].

## 3 Results

Eleven CRs participated, with various professional and cultural backgrounds, and who had investigation experiences ranging from 1 to 20 years. Their average age was 58, with a range of 35–71 years; seven were female. They had diverse experiences in investigations, ranging from leading teams, participating in the entire process, to combinations of various activities such as initial meetings, timeline planning, patient interviews and analysis meetings.

Ten focus groups were conducted with 26 health services staff in Victoria. The average age of participants was 49 years (range 36–68); 73% of participants were female. Eighteen of the 26 staff completed a demographic survey. Completion of the demographic survey was voluntary, and not all participants chose to complete it due to time constraints or varying degrees of engagement with the project. Those 18 people worked in nine different health services, with 89% working in a metropolitan health service. About three‐quarters (72%) of people worked in primarily non‐clinical roles, 92% of which were in Quality and Safety/Clinical Governance. Of those working in a clinical role, 60% worked in general medicine. All 18 staff had over 11 years' experience in the health system, with 72% having over 20 years' experience.

All participants' quotes below are labelled using a consistent identifier format as participant number and role (e.g., P1—CR; P6—Consumer Advisory Committee Member). This approach allows role clarity and identity anonymity.

Four high‐level themes were developed from the interviews and focus groups: formalising the CR role (including professional and personal qualities of CRs), investigation team‐level support (the chair and other teams), organisational integration (including processes and support) and health system‐wide cultural shift.

### 3.1 Theme 1: Formalising the CR Role: Personal and Professional Qualities of CRs

Both health service staff and CRs see a need to professionalise the CR role by defining its purpose and acknowledging its equal importance alongside other professional roles within the health system. This means a formal design and integration of the CR role in the organisational structure, establishing recruitment and retention programmes in place and creating pathways to attract and support suitable candidates.

…looking at a role and recruiting into a role would have a recruitment retention programme…. What are the channels that we can go out to…make people aware that they're available … with that clarity, not only of the roles, but the competencies and the development that you have to attach to that role… and then how would you onboard people … how would you bring them up to speed? My overarching feeling is that if we professionalise this [CR role] to a certain degree, if it's a role that we're going to continuously serve and is going to help us bring about better learning outcomes, then how do we apply the things to everything else?

P38, Consumer Advisory Committee Member

Equally important as other professional roles, the CR role must be grounded in principles of equity and inclusion, particularly by ensuring the voices and expertise of diverse communities, such as First Nations peoples, individuals with disabilities and other under‐represented groups. The cultural nuance can best be understood and articulated by members of these communities themselves.

…you're going into those more nuanced cultural things that you need that representation from those people that you know, I just think we have to work harder on getting that…

P6, CR

…the ideal way is to have a diverse group of consumers who are involved in those various operational facets throughout the hospital…

P9, CR

Defined professional and personal selection criteria should be set in the structured and formal recruitment process to ensure a good fit for the CR role. Care must be taken to ensure that such standardisation does not inadvertently exclude individuals with valuable lived experience who may not hold formal credentials. Key professional competencies include proficiency in English (in the case of Australia), strong health literacy and a solid understanding of the healthcare system. For example, both workshop participants and CRs note that investigation documents are often lengthy and complex, making it difficult to navigate them without adequate language skills.

…we have lists of glossaries, but understanding the language, you have to be prepared to question.

P10, CR

…when you get 100 pages of reading, which is not easy reading … it excludes a lot of people…

P6, CR

…you need a person who has both a certain level of actual English language literacy … as well as health system literacy … need to understand what you're reading and the discussions you're hearing, and health literacy … the ability to quickly learn…. It's not a high bar, but … it completely affects your ability to participate.

P39, Consumer Advisory Committee Member

Personal characteristics are equally crucial, including being able to draw on their personal experiences as a valuable source of knowledge, while giving due consideration to the information within the investigation. This should also involve demonstrating empathy and the ability to speak up respectfully and constructively in team discussions, even when navigating challenging power dynamics.

…to be able to speak up when you know you think you know something's off, something's not quite right here … that courage is probably the right word there.

P8, CR

…the right consumer … having a clear understanding of their role and how they can assist in the investigation is important … in context to the incident and how they, if they were in that incident, how we could have made things differently…

P20, Health Service Staff

…you have to be in a place where you can remove your personal emotion and your personal experience from the situation … the ability to interact respective, respectfully and non‐combatively … is part of that.

P39, Consumer Advisory Committee Member

…some baseline skills around, being confident speaking up, including in the face of pretty significant power imbalances, you need to be comfortable in a room where people don't necessarily want you there and aren't necessarily keen to listen to…it's a broad spectrum of personality.

P39, Consumer Advisory Committee Member

Additionally, CRs need to have the professional attributes to communicate complex issues with those with clinical training. There is a need for a combination of knowledge, personal characteristics and communication capacities and strategies.

…Some emotional skills are pretty useful, and particularly to be able to deal with the clinicians…. It's a very sensitive area when something's gone wrong in a hospital … it starts with empathy. You've gotta be able to put yourself in the position…

P11, CR

…the more obscure skills required … are the ability to be the voice of one in a discussion where you have a room full of clinically thinking people and you are the voice of one trying to draw their attention to other areas or other issues in relation to the incident.

P43, Consumer Advisory Committee Member

Like other professional roles, CRs should go through a rigorous application process to participate as a CR. It implies a move towards the professionalisation of the role, which will lead to outcomes where CRs should be equipped with the necessary skills, knowledge and attributes to meet the expectations of the investigation team. It also implies the need to design an inclusive recruitment process that recognises lived experience and avoids creating barriers for individuals from under‐represented communities, while ensuring that structured approaches are periodically reviewed to safeguard against unintentionally limiting diversity in the pool of CRs.

…consumers have to put in an expression of interest because it's advertised … write an application considering the terms of reference as well as the requirements of the health consumers recruitment process, and the applications are considered by an appointment group within health consumers…. It's quite a rigorous process … there are some steps required that you … meet with the chair, make sure you've got a package of papers about the committee before you join.

P43, Consumer Advisory Committee Member

### 3.2 Theme 2: Investigation Team‐Level Support

#### 3.2.1 Support From the Chair or Facilitator

Our participants emphasised the crucial role of the chair or facilitator on the patient safety investigation team, particularly in providing additional support and care outside of formal team meetings. Their approach can shape team dynamics, especially by empowering CRs and building their confidence in the investigation process. Such leadership requires sensitivity to the emotional dynamics involved in collaboration during patient safety investigations.

…it's important that the person who's chairing the meeting just simply gives you [CR] the opportunity to speak at the appropriate time.

P11, CR

Talking about the role of the chair, I think for a consumer [representative] it's great if there's a really good relationship with the chair, where the chair is open for you to contact them before the meeting to discuss any key issues that are coming up in relation to some of those patient papers.

P43, Consumer Advisory Committee Member

The chair should receive ongoing training co‐designed and based on the principles of working with CRs on how to facilitate investigations in a way that recognises their contributions. This is particularly important for those taking on the role, or working with CRs, for the first time. Without training and awareness of CRs, chairs may unintentionally overlook or undervalue their contribution, limiting the diversity of perspectives.

…make sure that you're [the chair] available to them [CRs] and they've got contact details for other members of the sponsorship for the review panel as well.

P31, Health Service Staff

…the ability to have a conversation one‐on‐one with the chair before your first meeting to talk about … what's going to be helpful for me? What might I need? … if you're doing this for the first time, about how to support the consumer role. The option to do that is really important for a consumer who wants it [to be a chair of investigations], and it shouldn't be assumed.

P38, Consumer Advisory Committee Member

There is a strong expectation that the chair or facilitator should set the tone for the investigation team during official meetings. Their behaviours can set a standard for how team members interact and engage with CRs. When the chair models respect and values CR contributions, it encourages others to follow suit. Conversely, a lack of such leadership may leave CRs feeling marginalised or undervalued, constraining their involvement.

It's the standard thing in facilitation of making sure that everybody is heard, and no one person dominates the discussion, that everybody's point of view is brought in and is respected and well heard.

P8, CR

It matters to set those expectations upfront and to have the chair speak positively about the consumer role from the beginning.

P39, Consumer Advisory Committee Member

#### 3.2.2 Other Investigation Team Members

Participants note that the support at the investigation team level is not solely about the chair/facilitator, but equally influential among other team members. Just as CRs should be elected based on defined criteria (such as lived experience, ability to represent community perspectives and communication skills), other team members (e.g., clinical staff and external subject matter experts) should also meet established requirements (such as relevant expertise, understanding of safety principles and a positive attitude towards CRs' involvement) before being appointed to the investigation team. This approach would ensure consistency in team capability and integrity of the investigation process. While some characteristics described overlapped with the attributes of CRs identified in Theme 1, the dynamics and interactions within the team are relational and contextual, which can shape the CR's engagement, influencing how inclusive and supportive the environment felt.

I think it should be selection criteria for the non‐consumer members of the team that they're basically told there will be a consumer rep on this group. You need to be OK with that. And you need to demonstrate that you're respectful and value that role to be on this team. And if you're not OK with it, get out.

P39, Consumer Advisory Committee Member

Both CRs and health service staff agree that support, inclusion and communication from the investigation team, before, during and alongside the official investigations are important to provide CRs with background knowledge about the specific adverse event. In particular, health service staff believe this approach would assist with a mutual understanding of CRs' benefits for the outcome of the investigation.

it's really to articulate what the consumer brings and to support that person to make sure that they can contribute … it's a two way…. It's about checking in with them during the support meeting, both before and after … anything that didn't sit well … we still need more work to do to … come to an agreed understanding of what the consumer does bring and how much benefit that brings…

P12, Health Service Staff

I think the initial support of providing all that information and that first debrief works quite well in terms of additional support … the post‐panel meetings debrief would be a good sort of suggestion.

P18, Health Service Staff

…when there's been an incident, often some organisations do a quick meeting that perhaps occurs two or three hours after the incident … really important that as the consumer, you are informed of what else happened outside of what was possibly documented, or it's been brought up.

P43, Consumer Advisory Committee Member

Team support can be formalised through mentorship or buddy programmes. It is important to ensure that CRs develop a basic understanding of medical terminology, while also helping clinicians recognise the contributions CRs bring, particularly the perspectives of patients and families. These programmes can help bridge knowledge gaps for CRs, both in relation to the investigations and broader health knowledge, while also encouraging clinicians to value the lived experiences and insights that CRs offer.

The best way I see that handled is when your paired up with a buddy person who's willing and has a good attitude, who's in the team and they say to you, look if you need to ask a practical question in the meeting to understand what's going on in the meeting, please ask it now.

P1, CR

It is also important for other team members to have aligned knowledge to work with CRs. For example, online training from SCV (see Box 1) is useful for both CRs and other team members to better understand the role. Additionally, practical exposure, such as observation of an investigation alongside official training, could complement the theoretical learning for developing skills and knowledge of the role.

…there would assist new consumers if they … lead in gently or even if they were not so much buddied but were able to observe. I mean that could even be quite an advantage for somebody just to sit in and observe and then be able to maybe go back to.

P4, CR

I did the SCV [Safer Care Victoria] training…. I was in a room full of health professionals…. I think there could be a little tweak to that training to help the consumer understand their role, and that would be quite beneficial for the health team to understand the consumer's role as well.

P7, CR

Box 1 **Victorian Model: Engaging CR in Patient Safety Reviews (Safer Care Victoria) [16].**

Purpose: To support the meaningful involvement of trained CRs in the review of adverse patient safety events across Victorian health services.

Key elements:

- Consumer voice: CRs bring independent patient, family and carer perspectives, helping to centre reviews around patient experiences.
- Selection and preparation: CRs are selected from a diverse pool via expression of interest, with opt‐out provisions based on personal experiences or sensitivities.
- Support structures: CRs receive orientation, logistical support, emotional well‐being resources and financial remuneration.
- Team integration: CRs participate fully in investigation activities, contributing to interviews, generating patient‐centred insights and co‐designing recommendations.
- Safeguards: confidentiality agreements, clear role definitions and escalation pathways protect CRs and ensure a respectful, inclusive review environment.

### 3.3 Theme 3: Organisational Integration: Support From Health Organisations

At the organisational level, participants believe that embedding the role of CRs within patient safety investigation teams requires alignment with health service governance and evaluation frameworks. This includes integrating CRs into clinical governance processes through dedicated pathways for escalation to resolve conflicts during investigations, while also recognising that the success of such integration depends on an organisational culture that genuinely values and supports consumer involvement, without which structural inclusion may not translate into meaningful empowerment.

Everybody who's in a team needs an escalation point beyond the chair or leader of that team when things start to go badly wrong, where you've got a culture problem or a psychological safety problem, or we're not prioritising a patient safety problem. You do need the contact details and willingness of somebody above … in the organisational structure that you can pull that emergency cord with…

P39, Consumer Advisory Committee Member

Such an approach of formalising the role of CRs should be developed in consultation with CRs, as they are better positioned to identify the types of support they need. Additionally, similar to other professional roles, CRs should have access to employee psychological support when required.

…what we can do better to prepare consumer reps through procedures and practises, whether this is where we need to be co designing … with the consumer representatives themselves … consumers can tap into their employee assistance programme if traumatised from about what they're hearing in a review.

P41, Consumer Advisory Committee Member

Participants also talked about enabling mechanisms to evaluate the effectiveness of the CR role and conducting regular reviews of CRs' impact and experiences in investigation processes at the organisational level. Ongoing evaluation and feedback imply organisational accountability so that the CR role will continuously improve.

Some measurements around the specific input that consumer representatives have put into the meeting or questions that they've asked, and whether they've been followed through. And around their recommendations and whether they've been addressed or taken up, or if not, the reasons for choosing not to do that.

P41, Consumer Advisory Committee Member

### 3.4 Theme 4: System‐Wide Cultural Shift: Support From the Broader Health System

Beyond structured procedures and organisational recognition, the meaningful inclusion of CRs requires a broader cultural shift led by boards, executives and senior leaders. While legislation and policy can set minimum standards, lasting changes depend on committed leadership to ensure consumer involvement is genuine rather than tokenistic.

This is about organisational culture coming from the top in terms of whether or not consumers are even included. That varies a bit now across states depending on legislative requirements, but whether they even aim for this and then how sincere they are in doing this, because in Victoria now it can be a bit like, we're supposed to.

P39, Consumer Advisory Committee Member

CRs should also be formally recognised at a national level through regulatory frameworks, as seen in the effectiveness of the Victorian model. While such recognition can support consistency and smoother coordination across jurisdictions, it must be complemented by strong organisational leadership to ensure that consumer involvement is authentic and not reduced to a compliance exercise.

At the state and national levels, CRs should be seen as valuable contributors who can move freely across jurisdictions. Developing a shared pool of trained CRs is essential to promote interstate collaboration and enable the sharing of resources across health services, regardless of jurisdictional boundaries. This approach would contribute to the establishment of a consistent, nationwide culture of engagement with CRs, which is particularly important for rural and regional health services that have limited access to skilled CR to draw on for patient safety investigations.

I think rural entities where … do an exchange programme between various health services for various reasons, rurality, specificity … these consumers become a resource of the state or even of the country, they're not a resource of the health service alone. The system needs to take some responsibility for resourcing … consumer reps need to be resources in a wider world rather than just resources of one health service. It's good for culture. It's good for people not being captured by health services.

P39, Consumer Advisory Committee Member

#### 3.4.1 Co‐Designed Best Practice Principles for Optimal Engagement With CRs on Patient Safety Investigations

Drawn from the research data and literature [15, 29, 30, 31, 32, 33, 34, 35, 36, 37, 38, 39], the research team and the consumer advisory committee worked together and co‐designed best practice principles for optimal engagement with CRs on patient safety investigations (Table 1). As part of our broader research, these principles will be implemented in selected investigation teams at chosen sites to assess their feasibility in real‐world settings.

**Table 1:** Best practice principles for health services to work with consumer representatives.

**(1) Formalise the CR role**
Professionalise the role of CRs by implementing a standardised and formal recruitment process that includes clearly defined selection criteria to ensure an appropriate fit for the role. This process should outline the required competencies and skills, as well as provide structured onboarding and training—similar to the approach used for other professional positions. This principle should be applied consistently across all CR roles within health services and health systems.
The recruitment of CRs should ensure broad representation across age groups, professional and socioeconomic backgrounds, cultural and linguistic communities, the LGBTQIA+ community, and individuals with disabilities or health conditions. Appropriate remuneration should be set in place.
CRs should possess a foundational level of health literacy, an understanding of the healthcare system and proficiency in English.
CRs should be able to draw on their personal experiences as a valuable source of insight, while also engaging thoughtfully with the information presented in the investigation. Key personal skills include empathy, the ability to speak up respectfully and the capacity to contribute constructively to team discussions—even when navigating complex or unequal power dynamics.
**(2) Provide investigation team‐level support**
Training programmes for CRs should cover core principles, methodologies, investigation processes and relevant state and national legislation. These programmes should be developed systematically and standardised across health institutions to ensure consistency and quality. In addition, training should include practical learning opportunities, such as shadowing an investigation or observing relevant healthcare services (e.g., dialysis units or surgical departments connected to the adverse event).
At the investigation team level, the chair or facilitator should be well‐trained and fully committed to supporting the meaningful engagement of CRs from the earliest stages of the investigation. They should model respectful and inclusive behaviour, actively affirming the CR's contributions. Importantly, the chair/facilitator should establish clear expectations that CRs are equal partners throughout the investigation, outline how each team member's expertise will be utilised, and promote the consistent use of plain language in all communications. A mentorship or buddy system should also be included to support new CRs in their early involvement.
**(3) Organisational integration of the role**
Develop training and awareness initiatives and implement them across healthcare services to emphasise the benefits and contributions of CRs within organisational structures. These initiatives should target both CRs and other team members to promote mutual understanding and respect. The intended outcome is to establish a consistent organisational expectation that CRs and other team members will collaborate closely and constructively on investigation teams.
At the organisational level, embed the role of CRs within governance and evaluation structures. This includes integrating CRs into clinical governance processes, establishing a dedicated channel for escalation in cases of conflict during investigations, implementing systems to measure and evaluate the effectiveness of the CR role, and conducting regular reviews of CRs' impact and experiences in investigation processes.
At the organisational level, provide the appropriate support for CRs to ensure their sustained engagement and well‐being, including establishing dedicated and flexible payment systems, offering accessible psychological support, fostering a sense of community among CRs and maintaining ongoing communication through newsletters or similar channels.
**(4) System‐wide cultural shift**
At the policy level (e.g., the departments of health and governments), take a leadership role and set enforceable standards and expectations regarding the involvement of CRs in investigation teams. These expectations should be supported by key performance indicators related to CR participation, with progress measured through mechanisms such as surveys and formal reviews.
Governments and the departments of health should take a leadership role in developing a shared pool of trained CRs. By doing so, they can help drive a cultural and structural shift that recognises the importance and necessity of CRs in patient safety investigations.

## 4 Discussion

We undertook interviews with CRs, focus groups with healthcare staff and a co‐design workshop with consumers. We found that there is a need to professionalise the CR role; this would include implementing a structured recruitment process that clearly defines professional and personal selection criteria to ensure an appropriate fit for the role. At the investigation team level, providing training on investigation fundamentals is required for both CRs and other team members, fostering an understanding of the benefits CRs bring. A mentorship or buddy programme would help facilitate mutual learning for CRs and other team members. At the organisational level, the CR role should be embedded within governance structures, with dedicated escalation pathways, and mechanisms for ongoing evaluation and feedback to support continuous improvement. While these principles represent best practice, their implementation should be adaptable to different organisational contexts, providing the underlying intent of meaningful and sustained consumer involvement is maintained. At the broader health system level, a consumer‐centred cultural shift is needed, along with the development of a pool of trained CRs to strengthen CR participation across jurisdictions.

Our findings reflect the increasing recognition of consumers and CRs as key partners in many health systems, both internationally [40] and in Australia [18, 19, 41, 42]. Similar to broader CRs in healthcare, there is a need for training and education, as well as the development of an inclusive culture [28, 43, 44, 45]. Our research echoed some commonalities with best practice principles for CRs in general proposed by Merner et al. [45], particularly regarding committed leadership, mutual respect, shared decision‐making, and diversity and equity in participation. These principles also inform support needs and structural enablers.

On patient safety investigation teams, CRs offer a valuable counterbalance to clinical and organisational perspectives by foregrounding consumer needs and lived experiences [15, 17]. Much of the literature on CRs is focused on longer consumer health service relationships [29]. However, unlike long‐term involvement in consumer advisory groups, participation in patient safety investigations requires distinct forms of engagement, often intense, time‐limited and emotionally complex [9, 46, 47, 48]. The intensity of dealing with responses to harm could amplify emotional labour and interpersonal interactions within the investigation teams, compared to other consumer engagement in various committees. Psychological safety is recognised in patient safety and is acknowledged within consumer engagement guidance, often as emotional or trauma‐informed safety [49, 50]. Our findings strengthen this literature by demonstrating that psychological safety is essential for CRs through structured training and support before, during and after the investigation.

While frameworks such as Safer Care Victoria's consumer partnership model provide broad guidance [8], our study found that CRs still felt uncertain about their role and were, at times, inadequately integrated into investigation processes. Some participants expressed concerns about not being heard or valued [15]. Similarly, a study in South Australia [39] found that CRs experienced confusion around their responsibilities and expectations, which led to hesitation and reduced confidence in contributing to discussions. These challenges point to the need to formalise the CR role through implementing a structured recruitment and orientation process. Such a process should include defined personal and professional selection criteria, such as demonstrated empathy, emotional intelligence and capacities to navigate power imbalance, health literacy and knowledge and effective communication. Aligning CR capacities with the specific demands of patient safety investigations can promote a better fit for the role, strengthen participation and ensure both the well‐being of CRs and the integrity of their contributions to the investigation process. Meanwhile, consistent with the broader consumer engagement literature [28, 43, 51, 52], a tension exists between the need for CRs who possess certain skills and capacities and the imperative to ensure inclusion, particularly of those from marginalised groups or people at greater risk of harm. This tension risks reinforcing existing power dynamics and tokenistic forms of involvement rather than accommodating a wider range of experiences and capacities. The idea of professionalism in CR roles must consider remuneration for fairness and transparency [15, 53, 54]. Appropriate payment recognises lived‐experience expertise and helps reduce economic barriers to participation for diversity and equity.

The structural design of CR roles within patient safety investigation teams, often characterised by short‐term engagement and limited clinical background, can lead to their contributions being undervalued or misunderstood by other team members [38]. These dynamics are frequently shaped by entrenched power imbalances between health professionals and CRs [37, 55, 56]. As mentioned earlier, investigation teams are convened and constrained for a specific purpose and operate within tight time frames. CRs' experiences within these teams are relational and contextual, shaped by team dynamics and interpersonal interactions. To ensure that CRs can participate meaningfully, it is essential to embed team‐level supports that promote mutual collaboration. This includes providing structured training not only for CRs but also for other team members, to build a shared understanding of the CR role, its purpose and the value of lived experience in investigations. In addition, the implementation of a buddy or mentorship programme can serve as a practical, low‐resource intervention to support CRs throughout the investigation process. Such programmes have been shown to reduce emotional distress [57] and promote reciprocal learning in other healthcare settings in the international literature [58, 59]. Given the typical duration of investigations (3–4 months), a sustained buddy arrangement could improve team cohesion, challenge hierarchical norms and create a more inclusive environment. By doing so, teams can establish clearer expectations for CR contributions [38] and foster a culture where the CR role is consistently recognised and respected within the investigation process [39].

At the organisational level, concerns about the tokenistic involvement of CRs remain prominent in the literature, often stemming from power imbalances within health services [41, 45, 56]. In some cases, consumers are perceived as resources to be drawn upon rather than equal partners in decision‐making [56]. Our findings similarly highlight that without structural integration, CRs' contributions to patient safety investigations may be overlooked or undervalued. Participants emphasised the need for dedicated escalation pathways, formal channels through which CRs can raise concerns or challenge investigation processes, as a critical mechanism to ensure their voices are heard and respected. When the CR role is not embedded within the organisational structure, CRs may feel discouraged from questioning some reviews or offering alternative perspectives, which in turn limits their ability to contribute meaningfully. To move beyond these institutional barriers [29], organisations must formally integrate the CR role by establishing clear reporting lines, dedicated support structures and continuous evaluation and feedback mechanisms. These actions will not only strengthen the legitimacy of the CR role but also support ongoing learning and improvement, helping to ensure CRs' participation in investigations is both effective and valued.

Patient safety is a complex domain, particularly when seeking to implement system‐wide changes that address deeply embedded professional, social and cultural norms [60, 61]. At the centre of effective investigation are principles of transparency, compassion and meaningful consumer engagement following adverse events [62]. Despite emerging recognition of the importance of consumer involvement, the evidence base for their sustained and structured participation in patient safety investigations remains limited [15, 63], reflecting the ongoing need for cultural transformation across health systems. Achieving such a shift requires not only a commitment to consumer‐centred care but also the promotion of CRs as valued contributors at all levels of the organisational hierarchy. This includes building awareness among health professionals and leaders, supported by legislative or policy levers that mandate and normalise CR involvement. Importantly, developing and maintaining a shared system‐wide pool of trained CRs can help ensure consistency, readiness and equity in access across health services. Together, these efforts can promote a culture where CRs are not only included, but actively recognised as integral to the integrity and responsiveness of patient safety investigations [64].

### 4.1 Strengths and Limitations

A key strength of this study lies in its triangulation of perspectives, incorporating the experiences and insights of CRs, health service staff and a consumer advisory committee. This methodological approach ensures that our interpretation of the data and the co‐designed principles derived from it are firmly grounded in the experiences of consumer engagement. Although primary data collection was conducted in a single Australian state (Victoria), our consumer advisory committee included members from four different states and territories, each bringing extensive experience and knowledge in patient safety investigations.

One limitation of our study is the limited pool of CRs that we could draw on, which constrained the diversity of our sample, even though we aimed to include more perspectives from culturally and linguistically diverse (CALD) communities and individuals from lower socio‐economic backgrounds. However, we gathered insights through discussions with CRs and health service staff about their experiences working with people from diverse backgrounds.

## 5 Conclusion

This is the first study that proposes a set of best practice principles to work with CRs on the patient safety investigation team, which were co‐designed with consumers across four jurisdictions. Key findings will inform strategies to better integrate CRs in the system: first, it is important to formalise the CR role, like other professional roles, with a rigorous recruitment process in place and clearly defined selection criteria, pathways, remuneration, ongoing education and retention programmes. Second, appropriate training of the fundamental principles of conducting investigations is required for both CRs and other team members. Ongoing support from the investigation team, particularly from the chair/facilitator and other team members before, during and alongside the investigation, is crucial to fostering mutual learning. A buddy programme would enhance the overall performance of the investigation process. Third, health organisations need to embed the CR role in the organisational hierarchy through demonstrated leadership commitment, implementing escalation channels, ongoing feedback and evaluation for continuous improvement. Finally, more consistent and systematic support from health services and broader health systems is essential to foster a cultural shift that values CRs' participation on patient safety investigation teams. Establishing a pool of trained CRs would help ensure a consistent and sustainable approach to their involvement across jurisdictions.

## Author Contributions

**Yinghua Yu:** conceptualisation, methodology, data curation, investigation, formal analysis, writing – original draft, writing – review and editing. **Peter D. Hibbert:** conceptualisation, methodology, data curation, investigation, formal analysis, supervision, funding acquisition, writing – review and editing. **Liat Watson:** data interpretation, writing – review and editing. **Jennifer Morris:** data interpretation, writing – review and editing. **Zoe Fernance:** data interpretation, writing – review and editing. **Jenny Berrill:** data interpretation, writing – review and editing. **Duncan Brown:** data interpretation, writing – review and editing. **Matthew Ames:** data interpretation, writing – review and editing. **Charlotte J. Molloy:** writing – review and editing, project management, validation. **Lorelle Bowditch:** writing – review and editing, validation. **Mia Bierbaum:** methodology, investigation, writing – review and editing, validation.

## Ethics Statement

The study was conducted in accordance with the Northern Sydney Local Health District (NSLHD) Human Research Ethics Committee (approval number: 2023/ETH02431).

## Consent

All patients were informed that participation was voluntary and provided written informed consent before the interview.

## Conflicts of Interest

The authors declare no conflicts of interest.

## Supporting information

Appendix 1_ COREQ_Checklist ‐ Consumer Representative Best Practice Principles.

Appendix 2 ‐ Demographic & survey focus groups.

Appendix 3 ‐ Interview schedule for consumers.

Appendix 4 ‐ Focus group guide.

Appendix 5 ‐ Consumer advisory committee workshop guide.

## Data Availability

All data are confidential due to ethical considerations.

## References

[1] P. D. Hibbert , C. J. Molloy , T. D. Hooper , et al., “The Application of the Global Trigger Tool: A Systematic Review,” International Journal for Quality in Health Care 28, no. 6 (2016): 640–649. 27664822 10.1093/intqhc/mzw115

[2] World Health Organization , Patient Safety Incident Reporting and Learning Systems: Technical Report and Guidance. 2020, WHO: Geneva, Switzerland.

[3] National Academies of Sciences , Crossing the Global Quality Chasm: Improving Health Care Worldwide (National Academies Press, 2018). 30605296

[4] World Health Organization , Global patient safety action plan 2021‐2030: towards eliminating avoidable harm in health care (World Health Organization, 2021).

[5] R. Schwendimann , C. Blatter , S. Dhaini , M. Simon , and D. Ausserhofer , “The Occurrence, Types, Consequences and Preventability of In‐Hospital Adverse Events—A Scoping Review,” BMC Health Services Research 18 (2018): 521. 29973258 10.1186/s12913-018-3335-z PMC6032777

[6] D. W. Bates , D. M. Levine , H. Salmasian , et al., “The Safety of Inpatient Health Care,” New England Journal of Medicine 388, no. 2 (2023): 142–153. 36630622 10.1056/NEJMsa2206117

[7] L. Bowditch , et al., “Do Patient Safety Incident Investigations Align With Systems Thinking? An Analysis of Contributing Factors and Recommendations,” BMJ Quality & Safety (2025): 019063. 10.1136/bmjqs-2025-019063 PMC13311954 40940142

[8] Safer Care Victoria , Sentinel Events Annual Report 2021‐22. Safer Care Victoria, Victoria, Australia, 2023.

[9] L. Ramsey , J. Hughes , D. Hazeldine , et al., “Humanising Processes After Harm Part 2: Compounded Harm Experienced by Patients and Their Families After Safety Incidents,” Frontiers in Health Services 4 (2024): 1473296. 39742113 10.3389/frhs.2024.1473296 PMC11685113

[10] M. Panagioti , K. Khan , R. N. Keers , et al., “Prevalence, Severity, and Nature of Preventable Patient Harm Across Medical Care Settings: Systematic Review and Meta‐Analysis,” BMJ 366 (2019): l4185. 31315828 10.1136/bmj.l4185 PMC6939648

[11] Clinical Excellence Commission , The NSW Health Incident Management Policy, 2020, Clinical Excellence Commission: NSW Australia.

[12] Patient Safety Learning , Patient Safety Incident Response Plans: An Analysis and Reflection by Patient Safety Learning. May, 2025.

[13] P. Trbovich and C. Vincent , “From Incident Reporting to the Analysis of the Patient Journey,” BMJ Quality & Safety 28, no. 3 (2019): 169–171. 10.1136/bmjqs-2018-008485 30337497

[14] Safer Care Victoria , Adverse Patient Safety Event Guideline 2023.

[15] P. D. Hibbert , Y. Yu , C. J. Molloy , et al., “Perceptions and Experiences of Consumer Representatives on Patient Safety Investigation Teams: A Qualitative Analysis,” Health Expectations 28, no. 3 (2025): e70281. 40304616 10.1111/hex.70281 PMC12042694

[16] Safer Care Victoria , Consumer Representatives on Adverse Event Reviews: A Guide for Health Services. 2019.

[17] M. Bierbaum , Y. Yu , C. J. Molloy , et al., “Decades of Failure to Prevent Harm to Patients—Where Are We Going Wrong?: A Mixed Methods Study of the Perspectives of Health Services Staff Across Australia and Internationally,” Frontiers in Health Services 5 (2025): 1645575. 40964190 10.3389/frhs.2025.1645575 PMC12436320

[18] M. DelDot , E. Lau , N. Rayner , J. Spinks , F. Kelly , and L. Nissen , “Consumer Involvement in the Design and Development of Medication Safety Interventions or Services in Primary Care: A Scoping Review,” Health Expectations 27, no. 6 (2024): e70092. 39552111 10.1111/hex.70092 PMC11570683

[19] A. E. Hall , J. Bryant , R. W. Sanson‐Fisher , E. A. Fradgley , A. M. Proietto , and I. Roos , “Consumer Input Into Health Care: Time for a New Active and Comprehensive Model of Consumer Involvement,” Health Expectations 21, no. 4 (2018): 707–713. 29512248 10.1111/hex.12665 PMC6117488

[20] Australian Commission of Safety and Quality in Health Care , *National Safety and Quality Health Service Standards*. 2017.

[21] P. D. Hibbert , L. Raggett , C. J. Molloy , et al., “Improving Health System Responses When Patients Are Harmed: A Protocol for a Multistage Mixed‐Methods Study,” BMJ Open 14, no. 7 (2024): e085854. 10.1136/bmjopen-2024-085854 PMC11227800 38969384

[22] A. Tong , P. Sainsbury , and J. Craig , “Consolidated Criteria for Reporting Qualitative Research (COREQ): A 32‐Item Checklist for Interviews and Focus Groups,” International Journal for Quality in Health Care 19, no. 6 (2007): 349–357. 17872937 10.1093/intqhc/mzm042

[23] Australian Bureau of Statistics, *Snapshot of Victoria*. 2022.

[24] S. Kvale and S. Brinkmann , Interviews: Learning the Craft of Qualitative Research Interviewing (Sage, 2009).

[25] J. Kitzinger , “Qualitative Research: Introducing Focus Groups,” BMJ 311, no. 7000 (1995): 299–302. 7633241 10.1136/bmj.311.7000.299 PMC2550365

[26] V. Braun and V. Clarke , “Using Thematic Analysis in Psychology,” Qualitative Research in Psychology 3, no. 2 (2006): 77–101.

[27] P. Slattery , A. K. Saeri , and P. Bragge , “Research Co‐Design in Health: A Rapid Overview of Reviews,” Health Research Policy and Systems 18, no. 1 (2020): 17. 32046728 10.1186/s12961-020-0528-9 PMC7014755

[28] T. Greenhalgh , L. Hinton , T. Finlay , et al., “Frameworks for Supporting Patient and Public Involvement in Research: Systematic Review and Co‐Design Pilot,” Health Expectations 22, no. 4 (2019): 785–801. 31012259 10.1111/hex.12888 PMC6737756

[29] I. M. Busch , A. Saxena , and A. W. Wu , “Putting the Patient in Patient Safety Investigations: Barriers and Strategies for Involvement,” Journal of Patient Safety 17, no. 5 (2021): 358–362. 32195779 10.1097/PTS.0000000000000699

[30] C. R. Wales , J. A. Lababedi , A. Coles , P. Lee , and E. Clarke , “Consumer Representative Experiences of Partnership With Health Workers in Australia,” Patient Experience Journal 8, no. 3 (2021): 64–78.

[31] C. Ehrlich , M. Slattery , and E. Kendall , “Consumer Engagement in Health Services in Queensland, Australia: A Qualitative Study about Perspectives of Engaged Consumers,” Health & Social Care in the Community 28, no. 6 (2020): 2290–2298. 32511875 10.1111/hsc.13050

[32] C. Adams and A. J. Brown , “Partnering With Healthcare: The Experiences of Consumer Representatives,” Patient Experience Journal 10, no. 1 (2023): 4–9.

[33] N. M. Ries , B. Johnston , and J. Jansen , “Views of Healthcare Consumer Representatives on Defensive Practice:‘We Are Your Biggest Advocate and Supporter Not the Enemy’,” Health Expectations 25, no. 1 (2022): 374–383. 34859547 10.1111/hex.13395 PMC8849368

[34] A. Chauhan , Consumer Engagement for Patient Safety: Strategies for Consumer Engagement for Culturally and Linguistically Diverse Consumers in Cancer Services (Macquarie University, 2023).

[35] N. Roberts , H. Jacmon , B. Scanlon , C. Battersby , P. Buttrum , and C. James , “How Can We Meet the Needs of Patients, Their Families and Their Communities? A Qualitative Study Including Clinicians, Consumer Representatives, Patients, and Community Members,” BMC Health Services Research 23, no. 1 (2023): 809. 37507758 10.1186/s12913-023-09814-9 PMC10385916

[36] M. Koutantji , et al., “The Patient's Role in Patient Safety: Engaging Patients, Their Representatives, and Health Professionals,” Journal of Patient Safety and Risk Management 11, no. 3 (2005): 99.

[37] J. Oakman , L. S. Cahill , S. Clune , et al., “Effectiveness of Health Consumer Representative Involvement in Implementation of Interventions to Change Health Professional Behaviour,” International Journal for Quality in Health Care 33, no. 1 (2021): mzaa164. 33325521 10.1093/intqhc/mzaa164

[38] J. McPhee , T. Warner , T. Cruwys , B. Happell , and B. Scholz , “‘They Don't Really Know Why They're Here’ Mental Health Professionals' Perspectives of Consumer Representatives,” International Journal of Mental Health Nursing 32, no. 3 (2023): 819–828. 36727283 10.1111/inm.13124

[39] A. E. Johnson , B. Beacham , C. Moretti , and J. Wishart , “Concerns About Being a a Health Consumer Representative: Results of a South Australian Study on Consumer Perspectives,” Australian Journal of Primary Health 12, no. 3 (2006): 94–103.

[40] World Health Organization , WHO Global Strategy on People‐Centred and Integrated Health Services: Interim Report. 2015.

[41] D. Luo , J. McGlashan , K. Lamprell , G. Arnolda , J. Braithwaite , and Y. Zurynski , “Perspectives of Cancer Consumer Representatives on Their Involvement in Healthcare Service Improvement: A Qualitative Study,” BMC Health Services Research 24, no. 1 (2024): 1324. 39482689 10.1186/s12913-024-11669-7 PMC11526679

[42] J. M. Payne , H. A. D'Antoine , K. E. France , et al., “Collaborating With Consumer and Community Representatives in Health and Medical Research in Australia: Results From an Evaluation,” Health Research Policy and Systems 9 (2011): 18. 21569591 10.1186/1478-4505-9-18 PMC3118959

[43] J. Ocloo and R. Matthews , “From Tokenism to Empowerment: Progressing Patient and Public Involvement in Healthcare Improvement,” BMJ Quality & Safety 25, no. 8 (2016): 626–632. 10.1136/bmjqs-2015-004839 PMC4975844 26993640

[44] K. L. Carman , P. Dardess , M. Maurer , et al., “Patient and Family Engagement: A Framework for Understanding the Elements and Developing Interventions and Policies,” Health Affairs 32, no. 2 (2013): 223–231. 23381514 10.1377/hlthaff.2012.1133

[45] B. Merner , L. Schonfeld , A. Virgona , et al., “Consumers' and Health Providers' Views and Perceptions of Partnering to Improve Health Services Design, Delivery and Evaluation: A Co‐Produced Qualitative Evidence Synthesis,” Cochrane Database of Systematic Reviews 3 (2023): 013274. 10.1002/14651858.CD013274.pub2 PMC10065807 36917094

[46] J. Kok , I. Leistikow , and R. Bal , “Patient and Family Engagement in Incident Investigations: Exploring Hospital Manager and Incident Investigators' Experiences and Challenges,” Journal of Health Services Research & Policy 23, no. 4 (2018): 252–261. 30027771 10.1177/1355819618788586 PMC6187500

[47] L. Ramsey , S. McHugh , R. Simms‐Ellis , K. Perfetto , and J. K. O'Hara , “Patient and Family Involvement in Serious Incident Investigations From the Perspectives of Key Stakeholders: A Review of the Qualitative Evidence,” Journal of Patient Safety 18, no. 8 (2022): e1203–e1210. 35921645 10.1097/PTS.0000000000001054 PMC9698195

[48] S. Wiig , P. D. Hibbert , and J. Braithwaite , “The Patient Died: What About Involvement in the Investigation Process?,” International Journal for Quality in Health Care 32, no. 5 (2020): 342–346. 32406494 10.1093/intqhc/mzaa034 PMC7299194

[49] Imperial College London , Patient and Public Involvement Trauma‐Informed Guidance for Organisations and Healthcare Professionals. 2024.

[50] R. O'Donovan and E. McAuliffe , “Exploring Psychological Safety in Healthcare Teams to Inform the Development of Interventions: Combining Observational, Survey and Interview Data,” BMC Health Services Research 20, no. 1 (2020): 810. 32867762 10.1186/s12913-020-05646-z PMC7456753

[51] J. Ocloo , S. Garfield , B. D. Franklin , and S. Dawson , “Exploring the Theory, Barriers and Enablers for Patient and Public Involvement Across Health, Social Care and Patient Safety: A Systematic Review of Reviews,” Health Research Policy and Systems 19, no. 1 (2021): 8. 33472647 10.1186/s12961-020-00644-3 PMC7816359

[52] G. P. Martin , “Representativeness, Legitimacy and Power in Public Involvement in Health‐Service Management,” Social Science & Medicine (1982) 67, no. 11 (2008): 1757–1765. 18922611 10.1016/j.socscimed.2008.09.024

[53] NSW Health , Consumer, Carer and Community Member Remuneration. 2023.

[54] Safer Care Victoria , A Guide to Consumer Remuneration 2024.

[55] A. Ramsay , P. Hartin , K. McBain‐Rigg , and M. Birks , “Advocating for Patient Safety: Power Dynamics in Nurse Advocacy Practice in Australia—An Integrative Review,” Collegian 32, no. 2 (2025): 84–99.

[56] B. Scholz , J. Bocking , C. Platania‐Phung , M. Banfield , and B. Happell , “‘Not an Afterthought’: Power Imbalances in Systemic Partnerships Between Health Service Providers and Consumers in a Hospital Setting,” Health Policy 122, no. 8 (2018): 922–928. 30017107 10.1016/j.healthpol.2018.06.007

[57] N. McCool , J. Reidy , S. Steadman , and V. Nagpal , “The Buddy System: An Intervention to Reduce Distress and Compassion Fatigue and Promote Resilience on a Palliative Care Team During the COVID‐19 Pandemic,” Journal of Social Work in End‐of‐Life & Palliative Care 18, no. 4 (2022): 302–324. 36129825 10.1080/15524256.2022.2122650

[58] K. Schrøder , T. Bovil , J. S. Jørgensen , and C. Abrahamsen , “Evaluation of ‘the Buddy Study’, a Peer Support Program for Second Victims in Healthcare: A Survey in Two Danish Hospital Departments,” BMC Health Services Research 22, no. 1 (2022): 566. 35477365 10.1186/s12913-022-07973-9 PMC9043887

[59] S. N. Asmuri , M. Kadar , N. A. Razaob , C. S. Chui , and H. F. Mohd Rasdi , “The Effectiveness of the Buddy Program Training Module to Enhance the Daily Living Function, Social Participation and Emotional Status of Older Adults in Residential Aged Care Homes,” PLoS One 19, no. 4 (2024): e0301544. 38568914 10.1371/journal.pone.0301544 PMC10990167

[60] C. Macrae and C. Vincent , “A New National Safety Investigator for Healthcare: The Road Ahead,” Journal of the Royal Society of Medicine 110, no. 3 (2017): 90–92. 28278394 10.1177/0141076817694577 PMC5349381

[61] C. Macrae , “Investigating for Improvement? Five Strategies to Ensure National Patient Safety Investigations Improve Patient Safety,” Journal of the Royal Society of Medicine 112, no. 9 (2019): 365–369. 31115260 10.1177/0141076819848114 PMC6823993

[62] G. Louch , et al., “How Were Patient Safety Incidents Responded to, Investigated, and Learned From Within the English National Health Service Before the Implementation of the Patient Safety Incident Response Framework? A Rapid Review,” Journal of Patient Safety 1097 (2023): 10. 10.1097/PTS.0000000000001349 PMC12266792 40341374

[63] Monash Institute of Health Services Research , Literature Review Regarding Patient Engagement in Patient Safety Initiatives. 2008, School of Public Health and Preventive Medicine, Monash University.

[64] M. Bierbaum , et al., “Consumers' Needs and Expectations From Patient Safety Investigations,” unpublished manuscript, September 15, 2025.

