# Supplementary material for: Best Practice Principles to Work With Consumer Representatives on Patient Safety Investigation Teams
Source: Health Expect. 2026 Jan 23;29(1):e70543. doi: 10.1111/hex.70543 (PMC12828782; doi:10.1111/hex.70543)
Supplement: Supplementary file 2 — Appendix 2 ‐ Demographic & survey focus groups. [file HEX-29-e70543-s003.docx]

# Appendix 2 Participant Information

**You have now been enrolled in the study.**

**Please answer the following demographic questions, followed by a short survey. Thank you!**

Name:

Phone Number

1. Which health service do you most often work for?
2. Which professional group do you most identify with? Administration

Allied Health- Occupational Therapy Allied Health - Physiotherapy


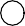

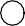

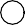

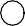

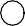

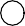

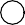

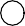

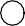

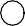

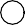

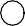

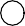

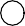

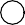

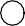

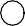


Allied Health - Speech Pathology Ancillary/Support Services Ambulance

Care Worker Dentist Dietitian Medical

Medical imaging diagnostic/technician Midwifery

Nursing Pharmacy

Radiation Therapist Scientist/Research Other

Please specify:

1. Is your role mainly clinical or non-clinical? Clinical (direct patient care)

Non clinical (indirect patient care and or ancillary/support services)


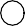

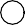


1. How many years have you been working in healthcare? 0-5 years

6-10 years


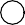

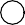

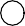

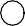


11-20 years

> 20 years

1. What is your primary work area? No specific unit Regional/facility office Anaesthetics/Recovery Emergency


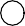

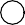

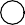

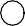

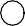

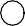

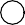

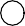

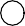

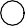

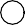

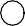

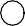

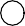

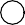

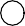

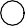


General Ward Intensive/Critical care Laboratory

Medicine (non-surgical) Mental health/psychiatry Obstetrics/gynaecology Oncology

Paediatrics Pharmacy Quality/Safety Radiology/Imaging

Surgery/Perioperative other

Please specify:

1. What best describes your role in the organisation? Executive

Senior manager Middle manager Line manager


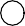

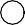

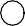

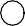

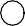

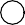


Team leader/supervisor Staff member

Gender: Male

Female Non-binary


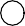

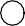

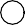

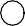

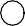


Prefer not to say

Prefer to use a different term

Please tell us the term you use to describe your gender:

Age:

Health Service you were recruited to this study from:

# Participant Survey

Please indicate your agreement with the statements below Thank you!

Please indicate your level of agreement with the following statements:

1. Investigations are a valuable use of resources for Strongly agree improving patient safety Agree

Neither agree nor disagree Disagree


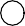

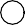

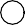

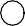

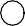


Strongly disagree

1. Actions taken in response to investigations prevent Strongly agree similar incidents from reoccurring Agree

Neither agree nor disagree Disagree


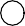

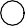

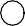

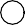

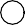


Strongly disagree

1. Healthcare organisations should be able to decide Strongly agree not to investigate an incident if no new information Agree

is likely to be gained for learning and improvement Neither agree nor disagree


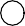

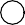

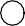

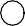

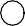


Disagree Strongly disagree

1. During investigations, clinicians are not unfairly Strongly agree

blamed for errors that may have occurred Agree


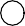

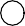

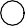

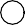

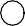


Neither agree nor disagree Disagree

Strongly disagree

1. Investigators have the right knowledge and skills Strongly agree

to undertake investigations and recommend change. Agree


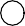

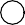

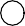

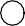

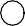


Neither agree nor disagree Disagree

Strongly disagree

1. Investigators have access to the right tools, Strongly agree

guidance, and support to conduct high-quality, Agree


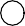

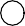

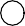

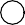

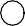


systems-based investigations. Neither agree nor disagree Disagree

Strongly disagree

1. Investigations consider when work processes and Strongly agree systems may have contributed to an incident. Agree

Neither agree nor disagree Disagree


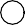

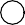

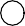

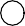

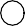


Strongly disagree

1. Investigators consider human factors and contextual Strongly agree factors that impact human performance. Agree

Neither agree nor disagree Disagree


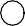

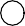

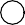

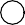

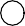


Strongly disagree

1. Investigations consider what went well, as well as Strongly agree what went wrong. Agree


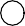

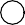

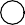

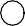

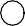


Neither agree nor disagree Disagree

Strongly disagree

1. Recommendations from investigations are aimed at Strongly agree the appropriate level (e.g., ward, department, Agree

hospital) of the healthcare system. Neither agree nor disagree Disagree


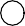

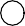

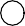

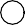


Strongly disagree

1. Recommendations from investigations are achievable Strongly agree

Agree

Neither agree nor disagree Disagree

Strongly disagree

1. Recommendations are accepted and supported by the Strongly agree organisation Agree

Neither agree nor disagree Disagree

Strongly disagree

1. Recommendations are reliably implemented Strongly agree Agree

Neither agree nor disagree Disagree

Strongly disagree

1. Recommendations lead to real improvements in Strongly agree patient safety Agree

Neither agree nor disagree Disagree

Strongly disagree

1. Learning from previous incidents prevents Strongly agree

reoccurrence or repeat incidents. Agree

Neither agree nor disagree Disagree

Strongly disagree

1. Lessons learned from incidents are communicated Strongly agree well across my organisation Agree

Neither agree nor disagree Disagree

Strongly disagree

1. Lessons learned from incidents outside my Strongly agree

organisation are shared with me Agree

Neither agree nor disagree Disagree

Strongly disagree

1. Learning from investigations drives continuous Strongly agree

improvement in patient safety. Agree

Neither agree nor disagree Disagree

Strongly disagree
