# Supplementary material for: Best Practice Principles to Work With Consumer Representatives on Patient Safety Investigation Teams
Source: Health Expect. 2026 Jan 23;29(1):e70543. doi: 10.1111/hex.70543 (PMC12828782; doi:10.1111/hex.70543)
Supplement: Supplementary file 3 — Appendix 3 ‐ Interview schedule for consumers. [file HEX-29-e70543-s004.docx]

Appendix 3: Semi-Structured Questions for Consumers

**Consumer representatives who have been involved in investigations**

- How many investigations have you been involved with?
- Describe your perception of the role of a consumer representative on the investigation.
- What tasks within the investigation have you generally undertaken?
  - (Prompts: development of timeline, interview questions, undertake interviews, writing up interviews, causation development, recommendation development, report writing, specialised contribution, e.g. human factors)
- What are the benefits of involvement in investigations of a consumer representative?
  - (Prompts: System-thinking, consumer-focused, recommendations are more likely to be implemented.)
- What are the weaknesses or risks?
  - (Prompts: Psychological distress, unhealthy, unmanaged conflict within the team, focus on blame, not feeling comfortable speaking up, uncertainty about the role, more difficult to organise or time-consuming)
- What are the barriers to being a consumer representative on an investigation?

I have some specific questions about the investigations in which you were involved.

- Were you involved in the creation of recommendations?
- After the conclusion of the investigation, did you see the final report?
- Did you feel you had sufficient psychological supports in place?
- Did you feel you could speak up?
- Prior to your role as a consumer representative, what information/training/support did you receive? Was it adequate?
- What support do consumer representatives need from the health service? What support do they need from Safer Care Victoria?
  - (Prompts: training, understanding the investigation methodology, medical jargon, counselling/support)
- What additional skills does a facilitator require if investigations include consumer representatives?
- Do you feel you provided a value add to the review? If so in what way?
- Would you recommend investigations include consumer representatives? Why?
- Would you do it again? Why/Why not?
