# Supplementary material for: Best Practice Principles to Work With Consumer Representatives on Patient Safety Investigation Teams
Source: Health Expect. 2026 Jan 23;29(1):e70543. doi: 10.1111/hex.70543 (PMC12828782; doi:10.1111/hex.70543)
Supplement: Supplementary file 4 — Appendix 4 ‐ Focus group guide. [file HEX-29-e70543-s001.docx]

**Appendix 4 Interview and focus group topic guide**

| 1. How do you decide now what to investigate and to what level? Aside from the official guidelines, what (in practice) influences this decision and why? (how could we do more with less?) |
| --- |
| 1. What structures do you have in place to decide? What skills and representation do you use? |
| 1. How might novel approached (RJC, Swarm huddle, Rapid Incident Review Meeting (RIRM) etc) change how level of incidents are determined? |
| 1. How are current practices working - In terms of getting the most value from the investigation process? Do you have any tools that you use to decide? |
| 1. IF there were no rules, what would you stop doing? what would you keep doing? How might this change the effectiveness and value of investigations or alter the use of resources? |
| 1. How would you prioritise the 12 criteria from the Grey literature review? – E.g., should severity be the only criteria? if not what else is important and in what order? |
| 1. If we were to develop a tool to help people with triage – who would use it and what features should it have to support each of those user groups? |
| 1. Do you routinely use other methods of detecting safety incidents? If so what other methods are in use and how does it compare to self-report? |
| 1. How does the Organisations safety and quality system and strategy prioritise resources for investigations and recommendations? (What % of resources are focused on investigating and responding to recs and what % are trying to implement solutions to known problems? Is there a strategy to look at high level problems based on multi-incident analysis? |
| 1. Do you believe the incident investigation method(s) used are effective for prompting change? 2. Are there investigation models or approaches that would work better? |
| 1. How would RJC approach be change the value and effectiveness of investigations?  (Psych. first aid, RCAs replaced with facilitated RJC review (forward-looking review of ‘the clinical care pathway’ not looking back from an incident), involved staff participate in the review, finding solution and sharing the learnings.) |
| 1. How can we learn from what goes right to make sure it happens more of the time in more places? |
| 1. Given the results of the investigation review – what should we do more or less of? what changes are needed to improve the quality of investigations or their recommendations |
| 1. Is the guidance for investigators is adequate? IF not, where is more support needed? |
| 1. What tips and tricks (" cookbook") advice would help panels to get through the investigation process more easily? What further advice or tools would help? |
| 1. How are actions tracked and monitored in each state – is this sufficient to understand the effectiveness and sustainability of recommendation? What else would help (e.g., AI) |
| 1. When do recommendations work? (effective/sustainable) when are they more likely to fail? |
| 1. What big changes are needed to help the health system respond better when patients are harmed (legal, cultural, political, financial, other?) |
| 1. How are recommendations currently implemented in your health service? Can you describe the process? Who is responsible for this process and what happens in practice? |
| 1. What feedback processes are currently in place in regard to implementation of recommendations? And how is success or failure of implementation of a recommendation measured? |
| 1. What do you think is working well with your recommendation implementation process? |
| 1. If you could change anything in order to make it easier to successfully implement recommendations what would this be? |
| 24. For VIC participants, have you worked with a consumer representative (CR) on the patient safety investigation team? What do you think work well and what’s not working well? What are the characteristics of CRs? What kind of support or structure needs to be in place to have better CR engagement? |
