# Supplementary material for: Best Practice Principles to Work With Consumer Representatives on Patient Safety Investigation Teams
Source: Health Expect. 2026 Jan 23;29(1):e70543. doi: 10.1111/hex.70543 (PMC12828782; doi:10.1111/hex.70543)
Supplement: Supplementary file 5 — Appendix 5 ‐ Consumer advisory committee workshop guide. [file HEX-29-e70543-s005.docx]

**Appendix 5 Consumer advisory committee co-design workshop plan (2 hours)**

**Acknowledgement of country**

**Introduction**/ purpose of the workshop **5 –10 mins**

Any general questions about draft outline? Then explain two parts of the workshop.

**Workshop activity 1 – Presentation for 10-15 min on general results (graphs). Reflections on our results 20-30 mins**

**Workshop activity 2 – Best practice principals**

Discussions on different themes **(20 mins each theme),** targeting specifically the discussions from activity 1. Use zooms white board to record best practice principles.

**Theme 1: Individual level – consumer representative**

Questions: What are the best practices at the individual level to optimize consumer rep’s engagement? Let’s think about the benefits, challenges and risks associated with being a consumer rep in the investigation, how we can better prepare new consumer rep to understand their roles.

**Theme 2: Investigation team level**

Questions: What are the best practices at the investigation team level to optimize consumer rep’s engagement? We would like to talk about different interactions with the chair/facilitator, clinicians, independent reviewers and other members of the investigation team, in what ways we can make changes to ensure consumer representatives are respected, heard and valued.

**Theme 3: Organizational level (e.g.: health services, LHD, HHS)**

**Questions:** What could organizations or leaders at the organizational level do to provide better support to engage with consumers? We can think about from a governance perspective, board executives, or organizational structure, how the inclusion of a consumer representative can be better fit into the organization. Let’s also be mindful of different organizational cultures, for example, in urban and rural settings, what we can do to better prepare consumer representatives through procedures and practices.

**Theme 4: Health systems (E.g. Safer Care Victoria, Clinic Excellence Commission, Department of Health Queensland, ACT Health)**

**Questions:** What could health services do to provide better support to engage with consumer reps? At the state level, if we are thinking about a cultural shift, how can we come up with better practices, policies or recommendations that could be effective from a top-down approach?

**Wrap up – 5 mins**

Let’s think out of the box and look beyond the four themes we just work on; is there any specific practice we can come up with?

Thanks to all participants for their engagement. The research team will work from here to draft the paper and take it to the next level. There will be drafts for comments and email exchanges later. We may require another workshop, but the majority will be done by email.
